# Database search engines and target database features impinge upon the identification of post‐translationally *cis‐*spliced peptides in HLA class I immunopeptidomes

**DOI:** 10.1002/pmic.202100226

**Published:** 2022-03-03

**Authors:** Michele Mishto, Yehor Horokhovskyi, John A. Cormican, Xiaoping Yang, Steven Lynham, Henning Urlaub, Juliane Liepe

**Affiliations:** ^1^ Centre for Inflammation Biology and Cancer Immunology (CIBCI) & Peter Gorer Department of Immunobiology King's College London London UK; ^2^ Francis Crick Institute London UK; ^3^ Max‐Planck‐Institute for Multidisciplinary Sciences Göttingen Germany; ^4^ Proteomics Core Facility, James Black Centre King's College London UK; ^5^ Institute of Clinical Chemistry University Medical Center Göttingen Göttingen Germany

**Keywords:** HLA, immunopeptidome, Mascot, PEAKS, peptide splicing

## Abstract

Unconventional epitopes presented by HLA class I complexes are emerging targets for T cell targeted immunotherapies. Their identification by mass spectrometry (MS) required development of novel methods to cope with the large number of theoretical candidates. Methods to identify post‐translationally spliced peptides led to a broad range of outcomes. We here investigated the impact of three common database search engines – that is, Mascot, Mascot+Percolator, and PEAKS DB – as final identification step, as well as the features of target database on the ability to correctly identify non‐spliced and *cis*‐spliced peptides. We used ground truth datasets measured by MS to benchmark methods’ performance and extended the analysis to HLA class I immunopeptidomes. PEAKS DB showed better precision and recall of *cis*‐spliced peptides and larger number of identified peptides in HLA class I immunopeptidomes than the other search engine strategies. The better performance of PEAKS DB appears to result from better discrimination between target and decoy hits and hence a more robust FDR estimation, and seems independent to peptide and spectrum features here investigated.

## 1 INTRODUCTION

CD8^+^ T cells patrol cells by scanning the sequence of peptides bound to Human Leucocyte Antigen class I (HLA‐I) complexes, which are present in thousands of different variants in the human population. The combination of binding affinity of peptides and HLA‐I variants as well as the avidity of T cell receptors αβ (TCRαβ) for peptide sequence is a highly efficient system to scan peptide‐HLA‐I complexes (Barbosa et al., 2021). Various techniques have been developed to identify peptides bound to HLA‐I complexes, that is, HLA‐I immunopeptidomes. A commonly used strategy is pulling‐down and eluting peptides from HLA‐I complexes, followed by measuring them through mass spectrometry (MS) and by analysing the data by applying exhaustive database search engines (Purcell et al., 2019). Despite the remarkable technical and bioinformatics progress in the field in the last decade, the choice of how eluting, measuring and analysing immunopeptidomes can strongly impinge upon the identified peptide pools. For instance, it has been shown that elution strategies affect peptide yields and create a bias in detected sequence repertoire (Nicastri et al., 2020). It is also generally accepted, and confirmed by various groups (Bichmann et al., 2019; Parker et al., 2021), that the number and features of identified peptides in canonical HLA‐I immunopeptidomes strongly vary depending on the search engines that are used in the analysis of MS measurements.

**FIGURE 1:**
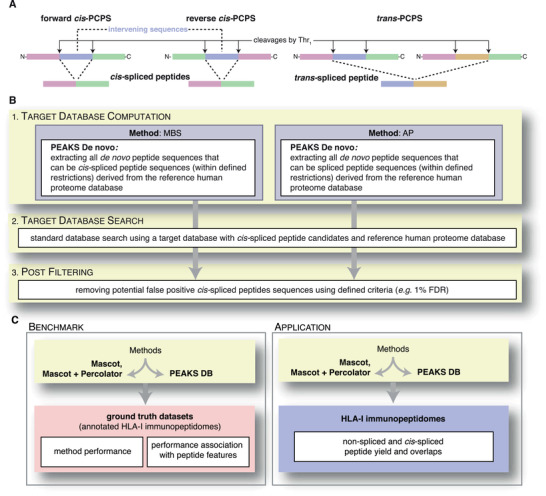
Proteasome‐generated spliced peptides and analysis workflow. (A) Proteasome‐generated spliced peptides can be formed by: i) *cis* PCPS, when the two splice‐reactants, that is, the non‐contiguous peptide fragments ligated by proteasomes, derive from the same polypeptide molecule; the ligation can occur in normal order, that is, following the orientation from N‐ to C‐terminus of the parental protein (normal *cis*‐PCPS), or in the reverse order (reverse *cis*‐PCPS); ii) *trans*‐PCPS, when the two splice‐reactants originate from two distinct protein molecules or two distinct proteins. (B) Overview of the workflow of MBS and AP methods. (C) Overview of the workflow used to test the performance of Mascot, Mascot+Percolator and PEAKS DB as final search engines

HLA‐I immunopeptidomes are mainly produced by proteasomes through the degradation of a broad range of proteins. Human cells can express various proteasome isoforms, which vary in conformation, catalytic dynamics, preference for substrates and preferential processing of peptide sequences. However, all proteasome isoforms can cleave after each known amino acid (Dianzani et al., 2019; Fabre et al., 2015; Guillaume et al., 2012; Kuckelkorn et al., 2019; Liepe et al., 2015; Mishto & Liepe, 2017; Mishto et al., 2014; Specht et al., 2020; Toste Rêgo & da Fonseca, 2019). These proteases can cut proteins and release ‘non‐spliced’ peptides, as well as ligate non‐contiguous peptide fragments, thereby producing spliced peptides. Proteasome‐catalysed peptide splicing (PCPS) can occur by combining non‐contiguous peptide fragments of the same molecule – i.e., *cis*‐PCPS – or of two distinct proteins ‐ i.e., *trans*‐PCPS (Liepe et al., 2018) (Figure 1A). Proteasome‐generated *cis*‐spliced epitopes were identified for the first time in 2004 (Hanada et al., 2004; Vigneron et al., 2004). They can target a CD8^+^ T cell response in vivo against bacterial antigens, which would be neglected by these T cells in the absence of *cis*‐spliced epitopes (Platteel et al., 2017). They can also activate CD8^+^ T cells through cross‐recognition of pathogen‐derived non‐spliced epitopes (Paes et al., 2019; Platteel et al., 2016). *Cis*‐spliced epitopes derived from melanoma‐associated antigens are recognised by CD8^+^ T cells in peripheral blood of melanoma patients (Ebstein et al., 2016; Faridi et al., 2020), and can be successfully targeted by adoptive T cell therapy in melanoma patients (Dalet et al., 2011; Robbins et al., 1994). *Cis*‐spliced epitopes could carry tumour‐specific mutations (Mishto et al., 2019, 2021) and seem to drive the immune response triggered by synthetic peptide vaccination in a mouse model of glioblastoma (Fidanza et al., 2021). Although *trans*‐spliced peptides are produced in vitro by proteasomes (Berkers et al., 2015; Dalet et al., 2010; Liepe et al., 2010; Mishto et al., 2012; Specht et al., 2020) and detected in HLA‐I immunopeptidomes (Faridi et al., 2018), their immunological relevance still need to be confirmed and thus they were not included in this study. In contrast, *trans*‐spliced peptides presented by HLA‐II molecules and produced by other proteases are immunologically relevant in type 1 diabetes (Arribas‐Layton et al., 2020; Delong et al., 2016; Reed et al., 2021; Wang et al., 2019).

Despite this seminal evidence of the immunological relevance of spliced peptides in HLA‐I immunopeptidomes, their frequency is still a controversial issue. After the publication of the first method for the identification of *cis*‐spliced peptides in HLA‐I immunopeptidomes (Liepe et al., 2016), other groups published alternative methods and obtained contrasting results about their frequency (Admon, 2021; Faridi et al., 2021; Mishto, 2020; Purcell, 2021). Two recent studies re‐analysed the list of *cis*‐spliced peptides, which we previously identified in HLA‐I spliced immunopeptidomes using Spliced Peptide Identifier (SPI) and SPI‐delta methods (Liepe et al., 2016, 2019), by using PEAKS X software. They identified many peptide‐spectrum matches (PSMs) that, according to their analysis (Erhard et al., 2020; Lichti, 2021), have been wrongly assigned to *cis*‐spliced peptides in the original papers. Since both SPI and SPI‐delta methods used Mascot as the database search engine (Liepe et al., 2016, 2019), Erhard and colleagues (Erhard et al., 2020) hypothesised that this phenomenon could in part be due to difference in PSM assignments by Mascot and PEAKS DB. Such hypothesis was shown to be correct for HLA‐I non‐spliced immunopeptidomes (Bichmann et al., 2019).

To verify whether this hypothesis was correct also for HLA‐I *cis*‐spliced immunopeptidomes, we here implemented two methods that, despite using a similar pipeline, reported a discordant range of *cis*‐spliced peptide frequencies in HLA‐I immunopeptidomes, that is, Bassani–Sternberg's method (MBS) (Mylonas et al., 2018) and Purcell's method (AP) (Faridi et al., 2018, 2019), and applied them using either Mascot or PEAKS DB as final search engine. In the case of Mascot, we also applied the Percolator post‐processing tool (i.e., Mascot+Percolator). Percolator is a semi‐supervised machine‐learning algorithm used to increase the number of peptides identified at a given FDR threshold and it is often used as post‐processing step of Mascot (The et al., 2016).

We tested Mascot, Mascot+Percolator and PEAKS DB on ground truth HLA‐I immunopeptidome datasets to compute search engine performance and to understand how performance is associated to the features of target databases, as well as on experimental HLA‐I immunopeptidome datasets to measure non‐spliced and *cis*‐spliced peptide yield.

## 2 MATERIALS AND METHODS

### 2.1 Cell lines

All cell lines were mycoplasma‐negative and cultured in 5% CO_2_ atmosphere at 37°C. K562‐B*07:02 and K562‐A*02:01 cell clones express single HLA‐I alleles. They derive from the leukaemia K562 cell line (ATCCCCL‐243), which does not express endogenous HLA‐I and ‐II molecules. The K562‐A*02:01 clone was generated as described elsewhere (Eichmann et al., 2014). Briefly, the HLA‐I allele was cloned and inserted into a pcDNA3.1 vector, transfected into K562 cell clone, which was then geneticin‐selected and periodically single‐cell sorted.

The K562‐B*07:02 clone was generated by Lorenz et al. (2017) as previously described, by linking cDNA gene sequences of HLA‐B*07:02 allele to GFP via an internal ribosomal entry site. This gene cassette was inserted into the γ‐retroviral vector MP71 for the generation of viral particles. Sorting of transduced K562 clones was performed upon surface HLA‐I expression using magnetic bead separation.

K562 clones were grown in RPMI medium with 10% FCS, 2 mM glutamine and PenStrep. The HLA‐A and HLA‐B allele data from Robinson et al. (2020) was appended to the transcriptome to control and validate the K562 cell clones.

The 721.221 HLA‐I deficient cell line are Epstein–Barr virus (EBV) transformed B cells, which do not express HLA‐I complexes. They have been transfected with an HLA‐A*02:01 expressing vector by Abelin et al. (2017), and they are here referred to as 721.221‐A*02:01.

### 2.2 RNA sequencing

RNA was extracted from K562, K562‐B*07:02 and K562‐A*02:01 cell line pellets (*n* = 1 per cell line clone) read by using the Qiagen ‘RNeasy Mini Kit’ and quantified using NanoDrop spectrophotometry. Extracted total RNA was sequenced and processed by GENEWIZ Inc. After polyA enrichment, mRNA was fragmented, and cDNA was produced using NEBNext Ultra RNA Library Preparation Kit with random priming. Sequencing was performed using HiSeq 2 × 150 PE HO with the depth of 20–25 million reads per sample. Reads were trimmed using Trim Galore with stringency parameter of 5. Quantification was performed using Salmon (v1.1.0) (Patro et al., 2017) with decoy‐augmented GENCODE v33 human reference transcriptome (Frankish et al., 2019). Salmon selective alignment mode was shown to improve the transcript quantification accuracy and can be used together with sample‐specific GC‐content, position and sequence bias models (Srivastava et al., 2019). In order to further enhance the sensitivity, particularly in short transcripts (Wu et al., 2018) the k‐mer size was reduced to 23 bp and 1,000 Gibbs samples were drawn from the posterior distribution of transcript abundances. To take advantage of Gibbs sampling and to correct for gene‐length bias, the tximport R package (Soneson et al., 2015) was used to import transcript quantification results and to scale the resulting transcript per million values using median transcript length amongst gene isoforms, and then the library size (dtuScaledTPM). Only the transcripts that have received more than 10 estimated counts in at least one sample were considered to be expressed and their GENCODE protein‐coding transcript translation sequences were selected for a common database for MS search.

### 2.3 Mass spectrometry

MS data generated for this project were collected using Orbitrap Fusion Lumos mass spectrometer coupled to an Ultimate 3000 RSLC nano pump (both from ThermoFisherScientific). Briefly, peptides were loaded and separated by a nanoflow HPLC (RSLC Ultimate 3000) on an Easy‐spray C18 nano column (50 cm length, 75 mm internal diameter; ThermoFisherScientific), coupled in‐line to a nano‐electrospray ionisation Orbitrap Fusion Lumos mass spectrometer (ThermoFisherScientific). Peptides were eluted with a linear gradient of 5%–45% buffer B (80% ACN, 0.1% formic acid) at a flow rate of 300 nl/min over 90 min at 50°C, with the exception of the MS file ‘PR487_Michele_20180604_B07.raw’, which was acquired under the same setting as above but over 60 min. The sample eluate was ionised by electrospray ionisation operating under Xcalibur v4.1. The instrument was first programmed to acquire using an Orbitrap‐Ion Trap method by defining a 3 s cycle time between a full MS scan and MS2 fragmentation using higher energy collision induced dissociation (HCD). We acquired one full‐scan MS spectrum at a resolution of 120,000 at 200 m/z with an automatic gain control (AGC) target value of 2 × 10^5^ ions and a scan range of 350–1550 m/z. The MS/MS fragmentation was conducted using HCD collision energy (30%) with an orbitrap resolution of 30,000 at 200 m/z. The AGC target value was set up as 5 × 10^4^ with a max injection time of 120 ms. A dynamic exclusion of 30s and 1–4 included charge states were defined within this method.

MS data previously published and derived from 721.221‐A*02:01 cell line clone were generated using an Orbitrap Q‐Exactive Plus mass spectrometer, as described by Abelin et al. (2017).

MS2 fragmentation spectra were recalibrated using the ‘Spectrum Files RC’ node in Proteome Discover. This calculates the PSM delta between theoretical and experimental mass in ppm and generates a mass shift curve. The optimal median value of calculated mass shift was then applied to the whole database search using either Mascot 2.7.01 or PEAKS X with a mass tolerance of either 5 ppm on precursor masses and 0.02 Da for fragment ions for the Orbitrap Fusion Lumos mass spectrometer, or 6 ppm on precursor masses and 20 ppm (0.03 Da when using PEAKS) for Q‐Exactive Hybrid–Quadrupole‐Orbitrap mass spectrometer. In the analysis with Mascot+Percolator, we used Percolator 3.0.5. The feature set for Percolator was more limited for these datasets than for a standard tryptic digest due to the non‐specific cleavage of proteasomes relative to trypsin and the fact that protein accession features were not applicable for de novo discoveries. The features used for Percolator were Mascot Ion Score, the percentage differences between the Ion Score of the PSM and the second and fifth highest ion scores for the same scan, the difference between the theoretical and experimental precursor mass and its absolute value, the length of the peptide, one hot encoding of the precursor charge, and one hot encoding of the post translational modifications which occurred in more than 10% of samples.

#### 2.3.1 Method workflows for the identification of *cis*‐spliced and non‐spliced peptides (Figure 1B)

We reproduced the workflow of methods published by Faridi et al. (2018) (AP method) and Mylonas et al. (2018) (MBS method). Both methods consist of two main steps: (i) generation of a target database that includes potential spliced peptide targets and (ii) identification of spliced peptides through application of a database search engine using a target database. AP and MBS methods apply PEAKS De novo to a reference human proteome database, and then make use of information gathered from de novo peptide sequencing to generate the target database. The target database is the combination of the reference human proteome database and the spliced peptide candidates computed via PEAKS De novo. AP and MBS methods generate the target database through different strategies. In their original publications, AP method employed PEAKS DB as final search engine, while MBS method employed the MaxQuant framework. Because we aimed to explore the difference between Mascot, Mascot+Percolator, and PEAKS DB, for each method we here applied either Mascot, Mascot+Percolator or PEAKS DB as final database search engine.

The implementation of the methods here applied and deviations from their original version, which were necessary either for technical reasons or to provide a consistent and comparable workflow for all methods, are the following:

**AP method** (Faridi et al., 2018): MS data was first searched against a reference human proteome database using PEAKS DB. Mass spectra not assigned as non‐spliced peptides with 1% FDR were searched using PEAKS De novo. For the following analysis, the top 5 de novo candidate sequences per MS2 spectrum with an Average Local Confidence (ALC) score larger than a computed cut‐off were exported. The ALC cut‐off was determined based on the ALC distribution of non‐spliced peptides, which were identified both via PEAKS DB at 1% FDR and PEAKS De novo. Among the top 5 de novo sequences within ALC scores above the cut‐off, all sequences were aligned to all possible non‐spliced peptides. If a potential non‐spliced peptide was detected, all respective de novo candidates were discarded from further analysis. Otherwise, if a potential *cis‐*spliced peptide was detected, all remaining respective de novo sequences were discarded, and the *cis‐*spliced peptide with the highest ALC score was extracted. If no potential *cis‐*spliced peptide sequence was amongst the de novo sequences, the sequence was aligned to all possible *trans‐*spliced peptides. Again, amongst all possible *trans‐*spliced peptide sequences the one with the highest ALC score was extracted. If non, *cis‐* and *trans‐*spliced sequences could not be found, the MS2 spectrum was not further considered. Finally, per MS2 spectrum, a maximum of one spliced peptide candidate (either *cis* or *trans*) was extracted. All extracted spliced peptide candidates were concatenated into in silico proteins, which were appended to the reference proteome database, thereby generating a target database. This target database was then used to re‐search the MS dataset using PEAKS DB. Identified peptides were extracted at 1% FDR as determined by PEAKS. To note, in our implementation, we used the PEAKS file ‘all‐de‐novo‐candidates.csv’, which Dr. Faridi confirmed being the actual file used in AP method (Faridi et al., 2018, 2020), rather than the ‘de‐novo‐only peptides.csv’, which was the file reported in Faridi et al. (2018).

**MBS method** (Mylonas et al., 2018): MS data were first searched against a reference human proteome database using PEAKS DB. MS2 spectra not assigned as non‐spliced peptide with PEAKS score ‐log10P larger than 15 were re‐searched using PEAKS De novo. The top five de novo candidate sequences per MS2 spectrum, were exported and aligned to all possible *cis‐*spliced peptides with an intervening sequence not longer than 25 amino acids (rather than 20 amino acids as in the original study). A PEAKS local confidence score (LCS) of at least 80 was required for each amino acid in the peptide candidates. Remaining de novo candidates that could be explained by *cis‐*peptide splicing were appended to the human reference proteome, thereby generating a target database. This target database was used to re‐search the MS data. Resulting peptides were filtered for 1% FDR. In their original study, Mylonas et al. (2018) employed Andromeda in the MaxQuant framework as their final search engine. In our study, we were not able to robustly analyse MS data in the open mzML or mzXML formats with MaxQuant. Furthermore, for the generation of the constructed ground truth datasets, MS1 and MS2 spectra from various MS runs were collected and merged into a single mzML file, which interfered with the indexing of those files, making them uninterpretable by MaxQuant. Therefore, MaxQuant could not be used for benchmarking, and we decided to exchange the Andromeda search engine in the MaxQuant framework either with the stand‐alone Mascot search engine or Mascot+Percolator (Mascot+Percolator). Both Andromeda and Mascot search engines operate on comparable algorithms, and analysis results were often found to be comparable in terms of precision and recall of non‐spliced peptides (Bichmann et al., 2019; Paulo, 2013). The search results were filtered for 1% FDR using a reversed decoy database, comparable to the implementations in MaxQuant. However, when applying MBS method to the HLA‐I immunopeptidomes, we filtered search results for 5% FDR, because Mascot has too low recall at 1% FDR.

#### 2.3.2 Spliced peptide candidate alignment to protein origin(s)

To determine if a sequence could be generated through splicing, it's possible splice reactants are aligned to all proteins in the reference proteome. A given peptide sequence is first split into two splice reactants, whereby we iterate over all possible splice sites in the candidate sequence. For a 9‐mer candidate sequence, we would generate all combinations of splice reactants with [1+8], [2+7], [3+6], [4+5], [5+4], [6+3], [7+2] and [8+1] amino acids of length. For each combination of splice reactants, we search the reference proteome for a matching sequence. If both splice reactants match to the same protein, the candidate sequence could be generated through *cis* splicing. If the two splice reactants match to two different proteins, the candidate sequence could be generated through *trans*‐splicing. If more than one possible explanation is found, AP and MBS methods implemented a hierarchy, which prefers non‐spliced over any spliced peptide and prefers *cis*‐ over *trans*‐spliced peptides.

#### 2.3.3 Generation of constructed HLA‐I immunopeptidome datasets and reference database for benchmarking framework (Figure 1C)

To determine the performance of Mascot, Mascot+Percolator and PEAKS DB within MBS and AP method framework, we applied the methods to ground truth datasets using constructed databases.

The strategy for the generation of a ground truth dataset of MS1 and MS2 spectra, that resembled the characteristics of HLA‐I bound peptides, followed the following steps: we used the MS RAW datasets of HLA‐I immunopeptidomes of two monoallelic cell lines (expressing either HLA‐B*07:02 or HLA‐A*02:01; three and six replicates, respectively) using Orbitrap Fusion Lumos and of the monoallelic HLA‐A*02:01 immunopeptidome dataset measured with Q Exactive Hybrid–Quadrupole (Abelin et al., 2017). The RAW MS data were analysed by both Mascot, Mascot+Percolator and PEAKS DB. The database used to obtain PSMs was the Uniprot reference proteome including isoforms (version 2016). Enzyme specificities were set to ‘unspecific’ in Mascot, Mascot+Percolator and PEAKS DB. Precursor mass tolerances were set to 5 ppm and 10 ppm for measurements on Fusion Lumos and Q Exactive Hybrid–Quadrupole, respectively. Fragment ion mass tolerances were set to 0.02 and 0.03 Da for measurements on Fusion Lumos and Q Exactive Hybrid–Quadrupole, respectively. PEAKS DB suggested peptides were filtered for 1% FDR using PEAKS’ internal decoy‐fusion strategy. Mascot suggested peptides were retained if the ion score was at least 30 and the *q*‐value was below 0.05. MS2 spectra assigned with the same sequence by both PEAKS and Mascot were extracted.

We then removed all peptides and MS2 spectra that were I/L redundant, thereby, we arrived at a final list of 1546, 1655 and 1246 peptides in Orbitrap Fusion Lumos for K562‐B:07*02 and K562‐A*02:0 and Q Exactive Hybrid–Quadrupole 721.211‐A*02:01 datasets, respectively. For each peptide, we selected exactly one MS2. If more than one MS2 spectrum was assigned to the same peptide sequence, we selected one MS2 spectrum randomly and discarded the remaining MS2 spectra.

The selected MS2 spectra and their corresponding MS1 spectra were merged in the mzML format to a new constructed HLA‐I immunopeptidome dataset. Resulting mzML files were tested for validity in both PEAKS DB and Mascot to check if all selected spectra were indeed correctly annotated (Table S1). The resulting three datasets represent the constructed ground truth HLA‐I immunopeptidome datasets and were employed in the benchmarking framework using constructed reference databases. The latter were generated by modifying the reference human proteome database so that the target sequences included in the benchmarking dataset could be identified only as one of the three categories: non‐spliced peptides, *cis*‐spliced peptides with intervening sequence shorter than 26 amino acids, or *trans*‐spliced peptides. Since only non‐spliced and *cis*‐spliced peptides could be identified by MBS method and we aimed to focus our analysis of the search engine performance in the identification of *cis*‐spliced peptides, we only considered non‐spliced and *cis*‐spliced peptides in the respective algorithm's performance analysis. Thereby, in the constructed HLA‐I immunopeptidome datasets, MS2 spectra derived from the pool of defined *trans*‐spliced peptides represented potentially high‐quality spectra, whose corresponding peptide sequence is not encoded in most database search strategies, and which could only be identified by exploring extremely large sequence search spaces. We considered those MS2 spectra as ‘trapping spectra’ and the corresponding peptides as ‘trapping peptides’. AP method may have an advantage when challenged with those trapping spectra, because it also considers the possible occurrence of *trans*‐spliced peptides. However, this potential advantage may come at the cost of overall less accurate peptide identification.

To produce a constructed reference database: (i) we modified the Uniprot standard proteome database by replacing all isoleucine (I) by leucine (L), that is, removing I/L redundancies that cannot be solved by MS; (ii) we split target sequences into the three peptide categories in equal proportion; (iii) in constructed reference database, we removed target sequences from the original reference database by replacing all substrings identical to a target peptide in the original reference database by a randomly sampled peptide sequence; (iv) we added non‐spliced, *cis‐* and *trans‐*spliced target sequences to the constructed reference databases by randomly sampling proteins from the reference database and appending the target sequences to either C‐ or N‐terminus of proteins. For *cis*‐ and *trans*‐spliced peptides we randomly sampled a splice‐site within each target sequence, split the target sequence into two splice‐reactants and append the splice‐reactants either to two different proteins (for *trans*‐spliced peptides) or to the same protein after including a random intervening sequence between the two splice‐reactants (for *cis*‐spliced peptides). After each iteration of appending, we tested that none of the other target peptide sequences could be assigned to a different peptide category.

#### 2.3.4 Benchmarking framework

To benchmark Mascot, Mascot+Percolator and PEAKS DB as final search engines in the constructed HLA‐I immunopeptidomes, we applied MBS and AP methods and extracted all identified PSMs using a predefined scoring schedule, covering a wide range of estimated FDRs. Briefly, when applying PEAKS DB for both methods we chose a range of 5–100 for the ‐log10P score. When applying Mascot as final search engine we varied the Mascot ‘peptide expect’ (pep_expect) value from 0 to 50. When applying Mascot+Percolator, we trained multiple percolator models to identify PSMs with Percolator *q*‐values corresponding to the ‘peptide expect’ values used to benchmark Mascot. We then extracted those PSMs identified with Percolator *q*‐value less than the corresponding thresholds and Percolator SVM scores greater or equal to 0.

PSMs for all methods and scoring threshold were extracted and stored in joint query tables, which merged the respective algorithm's PSMs with the known correct MS2 assignments. Those tables contained information about MS2 and peptide features, and analysed to investigate if they impinged upon Mascot, Mascot+Percolator and PEAKS DB performance.

The precision *P* of a method could be determined as the number of correctly identified PSMs over the number of all assigned PSMs for a given scoring threshold. Precision stood in direct relationship to the FDR, which is defined as *FDR = 1‐P*. The recall *R* of a method could be determined as the number of correctly identified PSMs over the number of all true PSMs.

#### 2.3.5 FDR estimation by Mascot, Mascot+Percolator and PEAKS DB

We extracted the number of assigned PSMs and estimated FDRs by Mascot, Mascot+Percolator and PEAKS DB for each scoring threshold. For both search engines, a target‐decoy approach to estimate FDRs (on PSM level) was employed. FDRs in PEAKS DB were estimated using a de novo assisted decoy‐fusion strategy (Tran et al., 2017, 2019). The decoy database was directly computed by PEAKS via reversing target sequences. Estimated FDRs were extracted manually for each scoring threshold from the PEAKS DB results summary report. When using Mascot as final search engine, we computed the decoy database by reversing all target sequences. The PSMs assigned by Mascot from this decoy database were used as negative training examples for Mascot+Percolator.

Both target and decoy databases were searched simultaneously. For each scoring threshold we counted the number of assigned target sequences (T) and assigned decoy sequences (D) and estimate the FDR as $FDR\;=\;100\frac{2D}{T+D}$.

#### 2.3.6 MS2 spectra characteristics

For each MS2 spectrum in our ground truth datasets we computed MS2 spectra characteristics (relative ion coverage and signal‐to‐noise ratio) and corresponding peptide characteristics (peptide length, hydrophobicity index).

### 2.4 Statistical analysis

All statistical analysis has been implemented in R. All statistics for performance measurement are described in the benchmarking framework. FDR calculation is described in the respective methods sections.

## 3 RESULTS

### 3.1 Evaluation of Mascot, Mascot+Percolator and PEAKS DB performance in *cis*‐spliced peptide identification in ground truth datasets

To evaluate the performance of Mascot, Mascot+Percolator and PEAKS DB as final search engines for identifying *cis*‐spliced peptides in HLA‐I immunopeptidomes, we implemented MBS and AP methods, using the three final search engine strategies. These two methods also generated target databases (FASTA files with canonical proteome and appended target spliced peptide sequences), which were used for the database search step (see also points 1 and 2 of Figure 1B). The target databases may have different features, which might impinge upon the final search engine performance.

In order to determine the methods’ performance in combination with either Mascot, Mascot+Percolator or PEAKS DB in terms of precision and recall, we applied them to ground truth datasets using constructed reference databases (see also Materials and Methods section).

The ground truth datasets were obtained from HLA‐I immunopeptidomes of HLA‐I mono‐allelic cell lines and were measured with either Orbitrap Fusion Lumos or Q‐Exactive Plus spectrometers to account for potential mass spectrometer bias. The HLA‐I immunopeptidomes were analysed for the identification of 8–15 amino acid long non‐spliced peptides in a standard immunopeptidomics workflow using both Mascot and PEAKS DB. PSMs that were assigned by both Mascot and PEAKS DB with high confidence and the same sequence to non‐spliced peptides were extracted and represented the ground truth datasets (Table S1).

The constructed reference databases were generated by modifying the reference human proteome database so that we knew which non‐spliced, *cis*‐spliced and trapping (unidentifiable) peptides were present in these ground truth datasets (see also Materials and Methods section). Since all target peptides were, in reality, non‐spliced peptide sequences derived from the canonical human proteins, we had to modify the reference database so that one‐third of the peptides could be identified only as non‐spliced peptides, one third only as *cis*‐spliced peptides (with intervening sequences shorter than 26 residues) and one third of the peptides could not be identified. The latter aimed to mimic the portion of HLA‐I immunopeptidomes that is neither non‐spliced nor *cis*‐spliced peptides, such as other potentially unknown peptide sequences not encoded directly in the human proteome database. In our benchmarking framework, we defined those unidentifiable peptides as *trans*‐spliced peptides, since these peptides could not be identified by MBS method. In our analysis, unidentifiable peptides represented the large pool of unconventional peptides – for example, those derived from alternative open reading frames (ORFs), intronic or intergenic regions, single amino acid mutations as well as *trans*‐spliced peptides (Erhard et al., 2020; Faridi et al., 2018; Laumont et al., 2016, 2018; Ruiz Cuevas et al., 2021) – and that cannot be identified with standard immunopeptidomics strategies. We chose this equal proportion of non‐spliced, *cis*‐spliced and unidentifiable peptides in the constructed reference databases bearing in mind the information gathered from the largest database of non‐spliced, *cis*‐spliced and *trans*‐spliced peptide products identified via MS in in vitro digestions of synthetic polypeptides (Specht et al., 2020), and some studies detecting other unconventional peptides in HLA‐I immunopeptidomes (Erhard et al., 2020; Faridi et al., 2018; Laumont et al., 2016, 2018; Ruiz Cuevas et al., 2021).

This strategy relying on constructed ground truth HLA‐I immunopeptidome datasets and cognate reference databases allowed a robust benchmark since we knew which non‐spliced, *cis*‐spliced and unidentifiable peptides were present in these ground truth datasets; thereby, we could directly compute precision and recall (PR) curves (Figure 1C).

**FIGURE 2:**
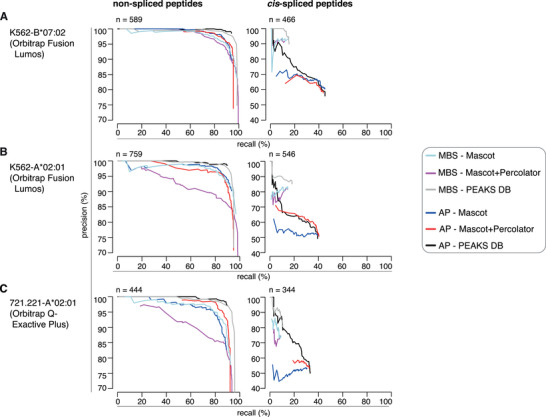
Performance of the three database search engine strategies for the identification of non‐spliced and *cis*‐spliced peptides in constructed ground truth HLA‐I immunopeptidome datasets. (A–C) Performance of the three database search engine strategies tested in the constructed ground truth HLA‐I immunopeptidome datasets of K562‐B*07:02 (A), K562‐A*02:01 (B) and 721.221‐A*02:01 (C). The original datasets were obtained through measurement by Orbitrap Fusion Lumos (A, B) or Q‐Exactive Plus (C). PR curves for the identification of non‐spliced and *cis* spliced peptides in constructed HLA‐I immunopeptidomes. PR curves report precision – i.e., number correctly identified peptides over number identified peptides – on the Y axis and recall – i.e., number correctly identified peptides over number correct peptides – on the X axis, are reported. Curves represent the performances by applying a range of scoring cut‐off. Number of true peptides present in each category is reported, which is a portion of the whole number peptides in the constructed HLA‐I immunopeptidome datasets of K562‐B*07:02 (*n* = 1556), K562‐A*02:01 (*n* = 1668) and 721.221‐A*02:01 (*n* = 1257)

We then applied AP and MBS methods – using either Mascot, Mascot+Percolator or PEAKS DB as final search engine – to each constructed ground truth HLA‐I immunopeptidome dataset and computed PR curves using a range of scoring cut‐offs. Both methods had high performance for the identification of non‐spliced peptides in all three constructed ground truth HLA‐I immunopeptidome datasets using both final search engines, with the exception of Mascot+Percolator in MBS framework. The latter may be the outcome of the limited number of PSMs in the constructed ground truth HLA‐I immunopeptidome datasets compared to the standard datasets on which Percolator is implemented (The et al., 2016). Within the range of high precision for the identification of non‐spliced peptides (i.e., 95% or more for recalls smaller than 80%), the application of Mascot and Mascot+Percolator consistently showed the worst performance especially in AP method and in the Orbitrap Q‐Exactive Plus dataset (Figure 2A–C). This outcome aligns with previous observations where PEAKS DB has identified significantly more higher confidence PSMs than Mascot+Percolator on immunopeptidome data (Bichmann et al., 2019).

**FIGURE 3:**
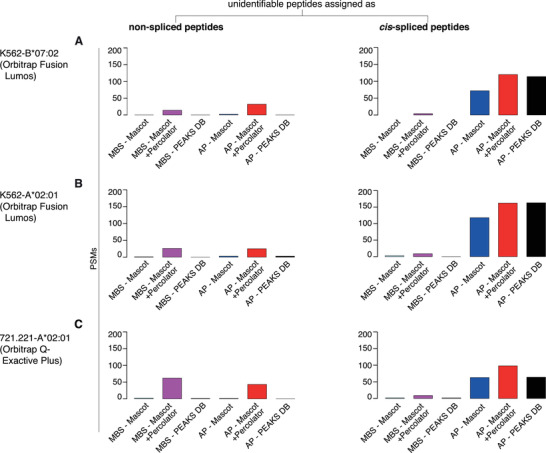
Misassignment of unidentifiable peptides included in the constructed ground truth HLA‐I immunopeptidome datasets. (A–C) Number of PSMs corresponding to unidentifiable peptides in the constructed ground truth dataset, which were wrongly identified as non‐spliced and *cis*‐spliced peptides by the various search engine strategies. The constructed ground truth datasets had 496, 539 and 463 unidentifiable peptides for K562‐B*07:02 (A), K562‐A*02:01 (B) and 721.221‐A*02:01 (C), respectively

By contrast, the method performances were strongly reduced in the identification of *cis*‐spliced peptides in all three constructed ground truth HLA‐I immunopeptidome datasets, as compared to non‐spliced peptide identification. The recall for the identification of these *cis*‐spliced peptides was limited, especially in the analysis carried out with MBS method. Also, overall, AP method showed a lower precision than MBS method. For both methods, precision of the identification of *cis*‐spliced peptides was strongly impaired by applying Mascot as the final search engine. Mascot performance in the identification of *cis*‐spliced peptides did not benefit from the addition of Percolator, with the exception of constructed ground truth HLA‐A*02:01 immunopeptidome datasets using AP method framework (Figure 2A–C). This difference in precision between peptide identification strategies was reflected in the misassignment of MS2 spectra that corresponded to unidentifiable peptides in the constructed reference databases. MBS method showed a similar number of unidentifiable peptides’ MS2 spectra wrongly assigned to non‐spliced and *cis*‐spliced peptide sequences, which was higher when we applied Mascot+Percolator as final search engine (Figure 3A–C). In contrast, AP method wrongly assigned more unidentifiable peptides’ MS2 spectra to *cis*‐spliced than non‐spliced peptide sequences (Figure 3A–C), which mirrored the lower precision of AP method, regardless of the search engine applied, in the identification of *cis*‐spliced peptides in the three constructed ground truth HLA‐I immunopeptidome datasets (Figure 2A–C).

### 3.2 The features of the target databases rather than the PSMs impinge on search engine performance

**FIGURE 4:**
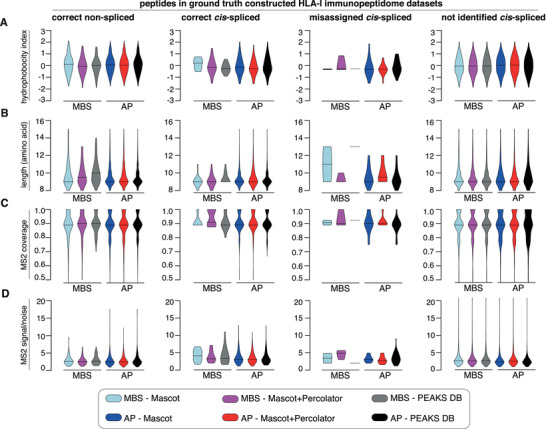
Performance of the three database search engine strategies in association with features of peptides and their MS2 in constructed ground truth HLA‐I immunopeptidome datasets. (A–D) The analysis was done by merging the results obtained by applying either Mascot, Mascot+Percolator or PEAKS DB within the framework of AP and MBS methods to the ground truth HLA‐I immunopeptidome datasets derived from whole HLA‐I immunopeptidomes of K562‐B*07:02, K562‐A*02:01 and 721.221‐A*02:01 cell lines. To have a dataset large enough to investigate peptides and MS2 features, peptides of all three constructed ground truth HLA‐I immunopeptidome datasets were analysed together. The analysed features are peptide hydrophobicity (A), peptide length (B), MS2 coverage (C) and MS2 signal‐to‐noise ratio (D). Peptides have been grouped based on their type – i.e., non‐spliced (A) and *cis*‐spliced peptides (B‐D) – and whether they were assigned with the correct sequence, misassigned or not assigned at all. Violin plots describe the value distribution. Median is depicted with a longitudinal line

MS2 spectra characteristics – such as ion coverage and signal‐to‐noise ratio – as well as peptide characteristics – such as length and hydrophobicity – may impinge upon both precision and recall of identified *cis*‐spliced peptides in HLA‐I immunopeptidomes. We, therefore, investigated the characteristics of PSMs and assigned *cis*‐spliced peptides, which may be associated with the poorer performance of Mascot compared to PEAKS DB in the constructed ground truth HLA‐I immunopeptidome datasets. None of the analysed characteristics seemed to be associated with better performance of PEAKS DB as compared to Mascot and Mascot+Percolator, as final search engine strategies (Figure 4).

**FIGURE 5:**
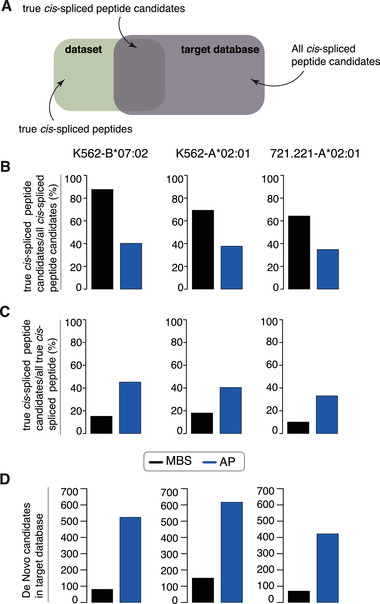
Features of target databases in constructed ground truth HLA‐I immunopeptidome datasets. (A) Definition of true *cis*‐spliced peptide candidates in target databases, all peptide candidates in target databases and all true *cis*‐spliced peptides in the three constructed ground truth HLA‐I immunopeptidome datasets. (B and C) Ratios of true *cis*‐spliced peptide candidates in target databases over all peptide candidates in target databases (B), and true *cis*‐spliced peptide candidates in target databases over all true *cis*‐spliced peptides in the three constructed ground truth HLA‐I immunopeptidome datasets (C). Target databases were generated via Peaks De novo by MBS and AP methods based on constructed reference human proteome database. (D) Number of De novo candidate sequences included in the target databases for each method and dataset

The different impact of the final search engines on each method's performance may be due, at least in part, to the features of the target databases, which are the combination of the reference human proteome database and the *cis*‐spliced peptide sequence candidates generated by both methods (see also points 1 and 2 of Figure 1B). To investigate this hypothesis, in the constructed ground truth HLA‐I immunopeptidome datasets and cognate target databases, we computed the number of true *cis*‐spliced peptide candidates, true *cis*‐spliced peptides and all *cis*‐spliced peptide candidates for both MBS and AP methods. These figures are specific for the dataset and the target database generated by MBS and AP methods, and are independent to the final search engine strategies. True *cis*‐spliced peptide candidates are *cis*‐spliced peptide sequences present in a target database, and in a constructed ground truth HLA‐I immunopeptidome dataset. True *cis*‐spliced peptides are *cis*‐spliced peptides present as such in a constructed ground truth HLA‐I immunopeptidome dataset. All *cis*‐spliced peptide candidates are all *cis*‐spliced peptide candidates present in a target database (Figure 5A). As a first analysis, we computed the ratio of true *cis*‐spliced peptide candidates over all *cis*‐spliced peptide candidates included in each method's target database for all three constructed ground truth HLA‐I immunopeptidome datasets. The higher this ratio, the more informed is the target database and, therefore, the easier it is to reach high precision, that is, the easier it is to identify true *cis*‐spliced peptide sequences (Figure 5B). Furthermore, we computed the ratio of true *cis*‐spliced peptide candidates included in each method's target database over all true *cis*‐spliced peptides in a constructed ground truth HLA‐I immunopeptidome dataset. The lower this ratio is, the more of the true target sequences are missing in a target database, which hinders their identification and, hence, results in low recall (Figure 5C). Finally, we analyse the size of the spliced peptide target databases for both, AP and MBS method (Figure 5D). The target database size is here defined as the number of De novo candidates that have been included as spliced peptide candidates in the final database search. According to our analysis, the number of true *cis*‐spliced peptide candidates in a target database represented a sizeable portion of the *cis*‐spliced peptide candidate in the same target database. MBS method, however, consistently showed a higher ratio of true *cis*‐spliced peptide candidates over all *cis*‐spliced peptide candidates in the target databases (Figure 5B). In addition, many true *cis*‐spliced peptides present in the constructed ground truth HLA‐I immunopeptidome datasets were overlooked by AP and, even more pronounced, by MBS methods (Figure 5C). Furthermore, the target databases for AP methods consist of approximately six times as many spliced peptides compared to the target database of MBS method (Figure 5D). These features of the target databases generated by both methods correlated well with the performance in the identification of *cis*‐spliced peptides in the constructed ground truth HLA‐I immunopeptidome datasets. Indeed, on the one hand, the method that had the highest ratio of true *cis*‐spliced peptide candidates over all peptide candidates in the target databases (Figure 5B) – that is, MBS method – had also the highest precision of the identification of *cis*‐spliced peptides in the constructed ground truth HLA‐I immunopeptidome datasets (Figure 2A–C). On the other, the method that had the highest ratio of true peptide candidates in a target database over all true peptides present in the constructed ground truth HLA‐I immunopeptidome datasets (Figure 5C) – that is, AP method – had the highest recall of the identification of *cis*‐spliced peptides in the constructed ground truth HLA‐I immunopeptidome datasets (Figure 2A–C).

The different features of the target databases containing both non‐spliced and *cis*‐spliced peptide candidates, and generated by the applied methods, might strongly influence the ability to distinguish true from false positive peptide sequence assignments and, hence, the FDR estimation by Mascot, Mascot+Percolator and PEAKS DB. This may impinge upon their performance as final search engine strategies both in constructed ground truth HLA‐I immunopeptidome datasets and in whole HLA‐I immunopeptidomes. To test this hypothesis, we analysed the association between assigned PSMs (which consisted of both true and false PSMs) and FDRs estimated by Mascot, Mascot+Percolator and PEAKS DB as final search engine strategies in whole HLA‐I immunopeptidome datasets eluted from K562‐B*07:02 and K562‐A*02:01 cancer cell lines and measured through an Orbitrap Fusion Lumos. We analysed these datasets through the application of AP and MBS methods by using either Mascot, Mascot+Percolator or PEAKS DB as final search engine strategies. In the analysis, we used a custom human proteome database based on K562‐B*07:02 and K562‐A*02:01 cell line RNA sequencing data (see Materials and Methods section). For a range of scoring thresholds, we extracted the number of all assigned PSMs and the corresponding estimated FDRs. A well‐performing search engine would assign a high number of PSMs at very low FDRs. The higher the number of assigned PSMs for low set and estimated FDR, the better is the search engine in discriminating true from false PSMs and the more sensitive is the search engine. We initially applied a standard pipeline using Mascot, Mascot+Percolator and PEAKS DB as final search engine strategies and the target databases including only non‐spliced peptides.

**FIGURE 6:**
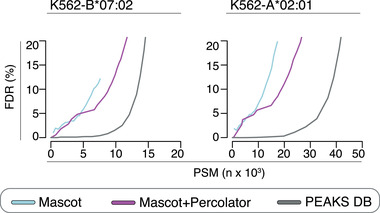
Association between identified non‐spliced PSMs and FDR estimation by applying different database search engine strategies to whole HLA‐I immunopeptidome datasets. PSMs identified for a range of estimated FDRs computed by applying either Mascot, Mascot+Percolator or PEAKS DB in a standard pipeline to K562‐B*07:02 and K562‐A*02:01 HLA‐I immunopeptidomes. The results have been obtained by applying the search engine strategies with target databases accounting only for non‐spliced peptides. The identified PSMs accounted for both true and false assignments

When applying PEAKS DB as final search engine the FDR‐PSMs curves were flat until a certain scoring threshold was reached, after which the estimated FDRs increased strongly (Figure 6). This allowed to determine a reliable scoring threshold for 1% FDR. On the contrary, when applying either Mascot or Mascot+Percolator as final search engine, the estimated FDRs increased steeply with increasing number of assigned PSMs, which hindered a reliable FDR estimation (Figure 6). Mascot+Percolator had a better FDR computation than Mascot alone. This is in line with previous studies investigating the impact of Percolator on Mascot performance in trypsin digestion samples. The FDR computation difference between Mascot and Mascot+Percolator in our HLA‐I immunopeptidomes seemed less striking than reported by other in trypsin digestions (Kall et al., 2007).

**FIGURE 7:**
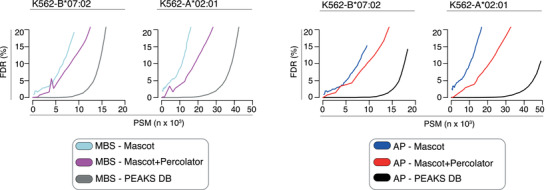
Association between identified non‐spliced and *cis*‐spliced PSMs and FDR estimation by applying different database search engine strategies to whole HLA‐I immunopeptidome datasets. PSMs identified for a range of estimated FDRs computed by applying either Mascot, Mascot+Percolator or PEAKS DB as final search engine of AP and MBS methods to K562‐B*07:02 and K562‐A*02:01 HLA‐I immunopeptidomes. The identified PSMs accounted for both true and false assignments. The results have been obtained by applying the methods and search engines with target databases accounting for both non‐spliced and *cis*‐spliced peptides

After this preliminary analysis, we applied MBS and AP methods using the generated target databases, which included both non‐spliced and *cis*‐spliced peptides, and either Mascot, Mascot+Percolator or PEAKS DB as final search engine strategy. In agreement with that shown in Figure 6, when applying PEAKS DB as final search engine the FDR‐PSMs curves were flat until a certain scoring threshold was reached, after which the estimated FDRs increased strongly (Figure 7), which allowed to determine a reliable scoring threshold for 1% FDR. By contrast, when applying either Mascot or Mascot+Percolator as final search engine strategies, the estimated FDR‐PSM curves showed similar behaviours to what was observed for the standard non‐spliced peptide identification pipeline (see Figure 6), which hindered a reliable 1% FDR estimation (Figure 7). Such a phenomenon could also indicate that Mascot and Mascot+Percolator may be less able to distinguish true from false PSM assignments in these kinds of samples combined with larger, non‐specific sequence search spaces.

For both MBS and AP methods, keeping a small estimated FDR, we have identified more PSMs by applying PEAKS DB as final search engine rather than either Mascot or Mascot+Percolator (Figure 7).

### 3.3 Identification of non‐spliced and *cis*‐spliced peptide through Mascot, Mascot+Percolator and PEAKS DB in whole HLA‐I immunopeptidomes

**FIGURE 8:**
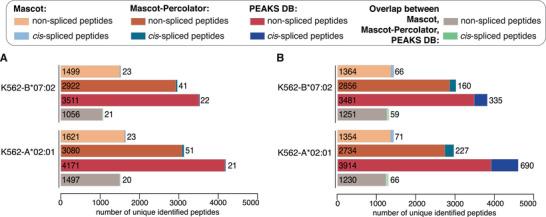
The computed frequency of non‐spliced and *cis*‐spliced peptides in K562‐B*07:02 and ‐A*02:01 HLA‐I immunopeptidomes depends on the database search engine strategy used. Number of unique 8–15mer long non‐spliced and *cis*‐spliced peptides identified by applying either Mascot, Mascot+Percolator or PEAKS DB as final search engine of either MBS (A) or AP (B) methods to K562‐B*07:02 and K562‐A*02:01 HLA‐I immunopeptidomes. The number of unique peptides identified through the different strategies is reported. The figures correspond to an analysis done by applying a 5% FDR for Mascot, 5% for Mascot+Percolator and 1% FDR for PEAKS DB

Since we estimated the performance of Mascot, Mascot+Percolator and PEAKS DB in identifying *cis*‐spliced peptides in constructed ground truth HLA‐I immunopeptidome datasets and the correlation of PSMs and FDRs in whole HLA‐I immunopeptidomes, we completed our study by applying the three database search engine strategies in AP and MBS method frameworks on the K562‐B*07:02 and K562‐A*02:01 HLA‐I immunopeptidome datasets. Because of the different FDR behaviour of Mascot, Mascot+Percolator and PEAKS DB (Figure 7), we applied the search engines by using 5% FDR for Mascot and Mascot+Percolator, and 1% FDR for PEAKS DB (Figure 8).

Within the framework of MBS method, the number of *cis*‐spliced peptides identified by applying the three final search engine strategies was similar. The frequency of *cis*‐spliced peptides (with intervening sequence smaller than 26 amino acids) varied between 0.5% and 1.6% (Figure 8A; Table [Link], [Link], [Link]).

By contrast, within the framework of AP method, which showed higher recall and lower precision of *cis*‐spliced peptides in constructed ground truth HLA‐I immunopeptidome datasets than MBS method (Figure 2), the number of *cis*‐spliced peptides identified by applying PEAKS DB was larger than that identified by applying either Mascot or Mascot+Percolator as final search engines. The frequency of *cis*‐spliced peptides (with intervening sequence smaller than 26 amino acids) varied between 4.6% to 15.0% (Figure 8B; Table [Link], [Link], [Link]). These frequencies of *cis*‐spliced peptides identified by the different implementations of MBS and AP methods were consistent with that published by the cognate research groups (Faridi et al., 2019, 2018, 2020; Mylonas et al., 2018).

For both MBS and AP methods, the overlap in identified non‐spliced peptides by applying either Mascot, Mascot+Percolator or PEAKS DB as the final search engine strategy was large, thereby suggesting that Mascot+Percolator and PEAKS DB confirmed the pool of non‐spliced peptides identified by Mascot and added to it a vast number of peptides (Figure 8A and B; Table [Link], [Link], [Link]). A similar behaviour was observed for *cis*‐spliced peptides within the framework of AP method (Figure 8B). The low number of identified *cis*‐spliced peptides by MBS method did not allow any conclusion with that method (Figure 8A). This hypothesis was confirmed at PSM level. For example, around 70% of the PSMs assigned as *cis*‐spliced peptides by AP method using Mascot–Percolator were assigned as such using PEAKS DB. In addition, most of the PSMs assigned as *cis*‐spliced peptides by AP method using PEAKS DB were not assigned to any peptide sequence using Mascot–Percolator as final search engine. Similarly, most of the PSMs assigned as non‐spliced peptides by AP method using Mascot–Percolator were assigned as such using PEAKS DB. In addition, half of the PSMs assigned as non‐spliced peptides by AP method using PEAKS DB were not assigned to any peptide sequence using Mascot–Percolator as final search engine (Table S5).

## 4 DISCUSSION

The analysis of constructed ground truth HLA‐I immunopeptidome datasets has unmasked the struggle of Mascot in identifying *cis*‐spliced peptides when applied as final search engine. The larger the reference database size, the lower the performance with Mascot. This emerged when we compared the performance of Mascot – and in part Mascot+Percolator – and PEAKS DB as final search engines of methods that have different overall performance and target database features for *cis*‐spliced peptides (Figures 2 and 5). The different performance between these three final search engine strategies did not seem to depend on either peptide and MS2 features or mass spectrometer (Figures 2 and 4). Rather, the analysis of the association between number of assigned PSMs and FDR in whole HLA‐I immunopeptidomes, hinted towards a more efficient FDR estimation algorithm of PEAKS DB compared to Mascot, which was only partially improved by adding Percolator to Mascot (Figures 6 and 7).

The outcome of this analysis might impinge upon the identification of other unconventional peptides in immunopeptidomics. Indeed, post‐translationally spliced peptides might be the vaster pool of unconventional peptides bound to HLA complexes but they are not the only one. Other PTMs are frequent in immunopeptidomes as well as cryptic peptides derived from, for example, putative non‐coding regions of the genome (Erhard et al., 2020; Laumont et al., 2018; Ruiz Cuevas et al., 2021). All unconventional peptide pools have the same inherent characteristic of enlarging the sequence search space compared to canonical non‐spliced peptides. Inevitably, this required search engines to accurately distinguish true PSMs from false PSMs due to potentially very high sequence similarity between true and false hits. Additionally, the larger a target database was, the lower the ratio of true peptide sequences over all entries in a target database, and hence it would be harder to identify true PSMs.

PEAKS DB reduced this issue through its de novo assisted decoy‐fusion strategy. PEAKS DB prefiltered the user‐provided reference database keeping only the top 8000 entries, which have a required number of de novo sequencing‐based sequence‐tags. This made not only the actual database search (PEAKS DB) efficient, but also reduced the final search space. Furthermore, PEAKS DB employed a decoy‐fusion strategy, whereby decoy sequences (inverted target sequences) were appended to each target entry in the database, thereby allowing for FDR estimation despite a two‐round search.

## CONFLICT OF INTEREST

The authors declare no conflict of interest.

## Supporting information

Supporting Information

Table S1

Table S2

Table S3

Table S4

Table S5

## Data Availability

Associated data The MS proteomics data that we generated have been deposited to the ProteomeXchange Consortium via the PRIDE (Perez‐Riverol et al., 2019) partner repository with the dataset identifier PXD031709. The Mascot, Mascot+Percolator and PEAKS DB search results tables are provided in Tables [Link], [Link], [Link], respectively. The HLA‐I immunopeptidome elution MS files published by Abelin et al. (2017) may be downloaded from MassIVE (http://massive.ucsd.edu) under the identifier MassIVE: MSV000080527. The data are directly accessible via ftp://massive.ucsd.edu/MSV000080527. The RNA sequencing data that we generated have been deposited in the NCBI Sequence Read Archive database with the accession code PRJNA721129.
