# Supplementary material for: Database search engines and target database features impinge upon the identification of post‐translationally cis‐spliced peptides in HLA class I immunopeptidomes
Source: Proteomics. 2022 Mar 3;22(10):2100226. doi: 10.1002/pmic.202100226 (PMC9286349; doi:10.1002/pmic.202100226)
Supplement: Supplementary file 1 — Supporting Information [file PMIC-22-0-s002.pdf]

Database search engines and target database features impinge upon the identification of post-translationally *cis*-spliced peptides in HLA class I immunopeptidomes

$ Correspondence to: michele.mishto@kcl.ac.uk, jliepe@mpinat.mpg.de.

Table S1. List of non-spliced peptides identified in HLA-I immunopeptidomes and used to create constructed ground truth HLA-I immunopeptidome datasets. Original source files and scan numbers, identified peptide sequences by Mascot and Peaks DB and their respective scores, detected retention times, annotated peptide types, and scan numbers in the resulting constructed ground truth dataset are provided. These spectra have been used to evaluate the performance of *cis*-spliced peptide identification methods in constructed ground truth HLA-I immunopeptidome datasets, derived from HLA-I immunopeptidomes eluted from K562-B*07:02, K562-A*02:01 and 721.221-A*02:01 cell lines. The table can be downloaded from the journal's portal.

Table S2. List of *cis*-spliced and non-spliced peptides identified in whole HLA-I immunopeptidomes by using Mascot as the final search engine for AP and MBS methods. Shown are all assigned PSMs in the format of the original Mascot search results tables using Mascot (5% FDR) as final search engine by AP and MBS methods. *Cis*-spliced peptides here reported could derive from sequences with an intervening sequence length not longer than 25 amino acid residues. Peptides have been eluted from HLA-I molecules of K562-B*07:02 and K562-A*02:01 cell lines. The table can be downloaded from the journal's portal.

Table S3. List of *cis*-spliced and non-spliced peptides identified in whole HLA-I immunopeptidomes by using Mascot+Percolator as the final search engine for AP and MBS methods. Shown are all assigned PSMs in the format of the original Mascot+Percolator search results tables using Mascot+Percolator (5% FDR) as final search engine by AP and MBS methods. *Cis*-spliced peptides here reported could derive from sequences with an intervening sequence length not longer than 25 amino acid residues. Peptides have been eluted from HLA-I molecules of K562-B*07:02 and K562-A*02:01 cell lines. The table can be downloaded from the journal's portal.

Table S4. List of *cis*-spliced and non-spliced peptides identified in whole HLA-I immunopeptidomes by using PEAKS DB as the final search engine for AP and MBS methods. Shown are all assigned PSMs in the format of the original PEAKS DB search results tables using PEAKS DB (1% FDR) as final search engine by AP and MBS methods. *Cis*-spliced peptides here reported could derive from sequences with an intervening sequence length not longer than 25 amino acid residues. Peptides have been eluted from HLA-I molecules of K562-B*07:02 and K562-A*02:01 cell lines. The table can be downloaded from the journal's portal.

Table S5. Overlap of peptides identified by using the three final search engine strategies in whole HLA-I immunopeptidomes. In Tabs 'shared identification by MBS' and 'shared identification by AP', we show the overlap between PSMs identified (or not identified) by using the three final search engine strategies with MBS and AP methods in the whole HLA-I immunopeptidomes eluted from K562-B*07:02

and K562-A*02:01 cell lines. In tabs ‘peptide identified by MBS’ and ‘peptides identified by AP’, we show the PSMs identified by MBS and AP, respectively, using the three final search engine strategies. The table can be downloaded from the journal’s portal.
